# Gamma SARS‐CoV‐2 variant of concern infection repercussions on pregnancy outcomes: A translational cohort study

**DOI:** 10.1002/ijgo.70985

**Published:** 2026-03-31

**Authors:** Guilherme M. Nobrega, Fabiana Granja, Mariene R. Amorim, Luciana S. Mofatto, Daniel A. Toledo‐Teixeira, Julia Forato, Priscilla P. Barbosa, Jose Paulo S. Guida, Adriana G. Luz, Giuliane J. Lajos, Carolina C. Ribeiro‐do‐Valle, Gisele A. Pedroso, Kamila C. S. Krywacz, Angelica Z. Schreiber, Giuliana D. Moreno, Rodrigo N. Angerami, Maria Luiza Moretti, Rodolfo C. Pacagnella, Renato T. Souza, Jose Guilherme Cecatti, Rodrigo G. Stabeli, Magnun N. N. Santos, Fernando R. Spilki, Ester C. Sabino, Nuno R. Faria, William M. de Souza, Jose Luiz Proenca‐Modena, Maria Laura Costa

**Affiliations:** ^1^ Department of Obstetrics and Gynecology, School of Medical Sciences University of Campinas Campinas São Paulo Brazil; ^2^ Laboratory of Emerging Viruses, Department of Genetics, Evolution, Microbiology and Immunology Institute of Biology, University of Campinas Campinas São Paulo Brazil; ^3^ Biodiversity Research Centre Federal University of Roraima Boa Vista Roraima Brazil; ^4^ Department of Clinical Pathology, School of Medical Sciences University of Campinas Campinas São Paulo Brazil; ^5^ Department of Internal Medicine, School of Medical Sciences University of Campinas Campinas São Paulo Brazil; ^6^ Campinas Department of Public Health Surveillance Campinas Brazil; ^7^ Oswaldo Cruz Foundation (Fiocruz‐SP) Ribeirão Preto São Paulo Brazil; ^8^ Department of Public Health Emergency Preparedness and Disaster, PAHO/WHO Brasilia Distrito Federal Brazil; ^9^ One Health Laboratory Feevale University Novo Hamburgo Rio Grande do Sul Brazil; ^10^ Tropical Medicine Institute, Medical School University of São Paulo São Paulo São Paulo Brazil; ^11^ Department of Infectious and Parasitic Disease, Medical School University of São Paulo São Paulo São Paulo Brazil; ^12^ MRC Centre for Global Infectious Disease Analysis J‐IDEA, Imperial College London London UK; ^13^ World Reference Center for Emerging Viruses and Arboviruses and Department of Microbiology and Immunology University of Texas Medical Branch Galveston Texas USA; ^14^ Hub of Global Health (HGH), University of Campinas Campinas Brazil

**Keywords:** COVID‐19, maternal health, morbidity, pregnancy, SARS‐CoV‐2, translational research

## Abstract

**Objective:**

This study conduct viral genome sequencing among severe acute respiratory syndrome coronavirus 2 (SARS‐CoV‐2)‐infected pregnant and postpartum individuals, and investigates disease severity and maternal and perinatal outcomes considering variant of concern (VOC) and non‐VOC groups.

**Methods:**

The study was designed as a prospective cohort study of unvaccinated pregnant and postpartum women with SARS‐CoV‐2 infection diagnosed by quantitative reverse transcription polymerase chain reaction between May 2020 and April 2021 at a maternal referral center. Initially, 111 participants were identified, and 50 presented with conditions for genome sequencing and further analysis. The SARS‐CoV‐2 genome was subjected to next‐generation sequencing in eligible cases. Sociodemographic characteristics, background, and clinical and obstetric outcomes were compared according to the SARS‐CoV‐2 genomic data (VOC vs. non‐VOC).

**Results:**

Among the sequenced cases, 18 were classified as VOC, among which 14 were Gamma cases and four Alpha. Oxygen desaturation, hospitalization, intensive care unit admission, invasive ventilation, and maternal death were significantly higher in SARS‐CoV‐2 VOC‐infected than in non‐VOC‐infected cases. All three cases of maternal death were classified as VOC, including two Gamma and one Alpha.

**Conclusion:**

Pregnant and postpartum people presented with an increased morbidity and mortality due to COVID‐19 caused by SARS‐CoV‐2 VOC, especially Gamma. The increased risk of clinical severity in the unvaccinated obstetric population underscores the importance of addressing the repercussions of the SARS‐CoV‐2 genomic evolution.

## 1 INTRODUCTION

The impact of severe acute respiratory syndrome coronavirus 2 (SARS‐CoV‐2) infection among pregnant and postpartum people was a concern from the beginning of the COVID‐19 pandemic,^1^ and later on with an increased awareness for the possibility of worse outcomes due to new SARS‐CoV‐2 variants of concern (VOCs).^2^, ^3^, ^4^, ^5^ In late 2020, four new SARS‐CoV‐2 VOCs were identified: Alpha (B.1.1.7), Beta (B.1.351), Delta (B.1.617.2), and Gamma (P.1), with Gamma spreading sharply over Brazilian locations, mostly related to a constellation of mutations in the Spike protein, promoting alterations in the immune response and increasing disease morbidity and mortality.^2^, ^6^, ^7^, ^8^, ^9^ The overall number of infected cases and deaths steeply increased in Brazil during the first months of 2021, with a recognized collapse of the health system in most regions, and most of the cases related to Gamma VOC.^6^, ^10^ The vaccination against SARS‐CoV‐2 started around early‐mid 2021 in Brazil, reducing the general disease clinical severity and shifting the previously outlined SARS‐CoV‐2 epidemiological scenario.^11^, ^12^, ^13^, ^14^

Thus, studies evaluating the clinical severity of SARS‐CoV‐2 VOCs during pregnancy and the postpartum period, particularly in referral centers that maintain full operational capacity, are key to elucidating the clinical impact of VOC infections. This is particularly relevant in regions where Gamma VOC was predominant during the first half of 2021, in contrast to the global predominance of Delta VOC during the same period. In this study, we investigated the clinical severity associated with VOC and non‐VOC infections during pregnancy and postpartum at a high‐complexity maternity referral center in Brazil, prior to the availability of SARS‐CoV‐2 vaccination, leveraging SARS‐CoV‐2 genomic data to assess the impact of Gamma VOC.

## 2 MATERIALS AND METHODS

### 2.1 Participants, respiratory samples collection and SARS‐CoV‐2 diagnosis

The present study is a 1‐year prospective cohort study conducted at the Women's Hospital at the University of Campinas (Unicamp), a referral maternity center in São Paulo state, Brazil, and the coordinating center of a large, multicenter, prospective cohort study on the effects of COVID‐19 during pregnancy (REBRACO, Brazilian Network of COVID‐19 in Obstetrics).^1^, ^15^ Samples were collected from SARS‐CoV‐2 symptomatic and asymptomatic (universal screening) individuals through a combined nasopharyngeal swab and disposed in sterile saline solution, immediately sent for diagnosis analysis, and stored at −80°C. All tests were performed by quantitative reverse transcription polymerase chain reaction (RT‐qPCR) assay for SARS‐CoV‐2 genes. Details of viral RNA extraction and SARS‐CoV‐2 RT‐qPCR are described in Methods S1. A total of 111 SARS‐CoV‐2‐unvaccinated pregnant or postpartum women who were admitted from May 2020 to April 2021 with confirmed SARS‐CoV‐2 infection were considered. These cases represent a cohort with further detailed evaluation of viral genome characterization. All participants received antenatal care and clinical management in accordance with established institutional protocols, including those for high‐risk obstetric conditions, irrespective of study group allocation.

### 2.2 SARS‐CoV‐2 genome sequencing and lineage classification and phylogenetic analysis

Positive samples by RT‐qPCR with cycle of quantification (Ct) values ≤28 were submitted for SARS‐CoV‐2 genome sequencing using the MinION platform (Oxford Nanopore Technologies, Oxford, UK)^16^ (Methods S2). All generated sequences are available at GISAID Initiative database (https://www.gisaid.org/); accession numbers are included in Table S1. The maximum‐likelihood (ML) phylogenetic tree was constructed using the genomes obtained and a compiled dataset of 130 sequences of VOC and non‐VOC lineages from Brazil, the UK, the USA, Italy, Portugal, and Spain, available on GISAID (Table S2). SARS‐CoV‐2 genome sequencing, lineage classification, and phylogenetic analysis information are described in Methods S2.

### 2.3 Clinical data

Clinical background, maternal SARS‐CoV‐2 infection characteristics, socio‐demographic information, and maternal and perinatal outcomes were retrieved from medical charts and further analyzed. The variables considered are described in Methods S3.

To further understand the role of such variants in clinical outcomes, we investigated the variables independently associated with VOC lineages using logistic regression and a decision tree model.

### 2.4 Epidemiological analysis

Local data on the number of reported SARS‐CoV‐2 infection cases in the considered period were compared to regional and country‐level data, to understand COVID‐19 dynamics regarding case incidence and local perspective. Detailed demographic, epidemiological, and clinical variables considered are described in Methods S4.

### 2.5 Group allocation and data analysis

For the cohort analyses, participants were allocated to analysis groups according to the SARS‐CoV‐2 variant identified through genomic sequencing. Cases were categorized as infection with a SARS‐CoV‐2 VOC or non‐VOC lineage, as described in the previous section “SARS‐CoV‐2 genome sequencing, lineage classification, and phylogenetic analysis.” Comparisons between groups were performed using odds ratios with 95% confidence intervals (CI) and the *χ* ^2^‐test or Fisher's exact, for expected values lower to 5, using Statistical Analysis System 9.2 and EPInfo 7.2.4 (CDC, USA). Simple logistic regression analysis was used to study factors independently associated with SARS‐CoV‐2 VOCs (sociodemographic, clinical background, obstetric characteristics and outcomes, possibly associated with SARS‐CoV‐2 infection and/or severity), performed with SAS System for Windows (Statistical Analysis System), version 9.2. (SAS Institute Inc., 2002–2008, Cary, NC, USA). The decision tree model was performed using R software v4.1.1 in all steps using *rpart* packages for the decision tree. Clinical data from 70% of the considered sample randomly selected was used to create a training set, and the remaining 30% of cases were used to test for accuracy, sensitivity, and specificity. A *P*‐value ≤0.05 was considered significant.

### 2.6 Ethical considerations

The research project followed all recommended rules for the use of human samples and was approved by the Research Ethics Committee of Unicamp, IRB #31591720.5.0000.5404. The recruitment period for this study commenced on May 1, 2020, and ended on April 17, 2021. All participants or those legally responsible for participants signed an informed consent form authorizing collection, storage, and use of clinical samples. This study is reported according to the STROBE statement.

## 3 RESULTS

In this study, of the 111 cases considered, 58 presented an RT‐qPCR Ct >28 for any SARS‐CoV‐2 target gene and, therefore, were ineligible for genome characterization. Fifty‐three patients were eligible and selected for genome sequencing (Ct ≤28 for SARS‐CoV‐2 for all target genes). Of these, three cases had low sequencing assay coverage (<45%) and were unable to perform genome classification. Then, we obtained 50 SARS‐CoV‐2 nearly complete genomes, of which 32 cases were classified as non‐VOCs and 18 cases were VOCs according to the Pangolin COVID‐19 Lineage Assigner tool and subsequent phylogenetic analysis (Figure S1). All VOC lineages of SARS‐CoV‐2 detected in this study had most of the signature mutations described for Gamma (P.1) VOC, followed by Alpha (B.1.1.7) (Tables S2 and S3).

A detailed description of the general characteristics of VOC (*n =* 18) and non‐VOC (*n =* 32) cases is presented in Table 1. Among the VOC and non‐VOC cases, both groups were mostly similar for reported underlying sociodemographic conditions and comorbidities. All SARS‐CoV‐2 infection symptoms were assessed at hospital admission, and the frequency of asymptomatic infection was not significantly different between the groups (Table 1).

**TABLE 1:** Comparison of sociodemographic characteristics, clinical and obstetric background, and COVID‐19 symptoms, according to the group of genomes: Variant of concern (VOC) versus non‐variant of concern (non‐VOC).

	VOC (*n =* 18)		Non‐VOC (*n =* 32)		Odds ratio (95% CI)	*P*‐value
General characteristics						
Age						
≤19	1	5.6%	0	0.0%	—	0.566
20–35	14	77.8%	23	71.9%		
≥ 36	4	22.2%	9	28.1%		
Skin color						
White	12	66.7%	21	65.6%	1.05 (0.3–3.6)	0.941
Non‐White	6	33.3%	11	34.4%		
Marital status						
With partner	11	61.1%	5	15.6%	**8.49 (2.2–32.6)**	**<0.001**
Without partner	7	38.9%	27	84.4%		
Schooling						
None or primary incomplete	1	5.6%	1	3.1%	—	0.085
Primary or secondary	17	94.4%	25	78.1%		
College or more	0	0.0%	6	18.8%		
Parity						
0	5	27.8%	9	28.1%	—	0.980
1–2	10	55.6%	17	53.1%		
≥3	3	16.7%	6	18.8%		
Singleton pregnancy	16	88.9%	29	90.6%	0.83 (0.1–5.5)	1.000
Twin pregnancy (two fetuses)	2	11.1%	3	9.4%	1.21 (0.2–8.0)	1.000
Comorbidities						
Diabetes mellitus	5	27.8%	9	28.1%	0.98 (0.3–3.6)	0.979
Chronic arterial hypertension	5	27.8%	4	12.5%	2.69 (0.6–11.7)	0.253
Obesity	5	27.8%	4	12.5%	2.69 (0.6–11.7)	0.253
Asthma	1	5.6%	1	3.1%	1.82 (0.1–31.0)	1.000
SARS‐CoV‐2 infection symptoms						
Fever	9	50.0%	15	46.9%	1.13 (0.4–3.6)	0.832
Cough	12	66.7%	17	53.1%	1.77 (0.5–5.9)	0.352
Rhinorrhea	7	38.9%	16	50.0%	0.64 (0.2–2.1)	0.449
Odynophagia	7	38.9%	6	18.8%	2.76 (0.8–10.1)	0.180
Headache	6	33.3%	15	46.9%	0.57 (0.2–1.9)	0.352
Myalgia	9	50.0%	14	43.8%	1.29 (0.4–4.1)	0.670
Dyspnea	7	38.9%	8	25.0%	1.91 (0.6–6.6)	0.304
Vomiting	2	11.1%	3	9.4%	1.21 (0.2–8.3)	1.000
Diarrhea	1	5.6%	0	0.0%	—	0.360
Anosmia	4	22.2%	11	34.4%	0.55 (0.1–2.1)	0.368
Ageusia	5	27.8%	10	31.3%	0.85 (0.2–3.0)	0.797
Asymptomatic	2	11.1%	3	9.4%	1.21 (0.2–8.0)	1.000

*Note:* Bolded *P*‐values indicate statistical significance (*P*‐value < 0.05).

The onset of SARS‐CoV‐2 infection occurred mainly in the third trimester of pregnancy, and the time between symptom onset and laboratory diagnosis was mainly prior to 7 days of symptoms in VOC and non‐VOC cases (Table 2). Even with overall high frequencies of preterm births (50.0% in VOC vs. 27.6% in non‐VOC, *P*‐value = 0.120) and c‐sections (72.2% in VOC vs. 58.6% in non‐VOC, *P*‐value = 0.346) among VOC cases, pregnancy outcome comparison results were not significantly different between the VOC and non‐VOC groups (Table 2).

**TABLE 2:** Comparison of clinical severity, maternal and perinatal outcomes of cases according to the group of genomes: Variant of concern (VOC) versus non‐variant of concern (non‐VOC).

	VOC (*n =* 18)		Non‐VOC (*n =* 32)		Odds ratio (95% CI)	*P*‐value
Clinical severity						
O_2_ saturation < 95%	8	44.4%	1	3.1%	**24.80 (2.8–223.3)**	**<0.001**
Hospitalization	14	77.8%	11	34.4%	**6.68 (1.8–25.3)**	**0.003**
ICU admission	6	33.3%	1	3.1%	**15.50 (1.7–142.6)**	**0.006**
Invasive ventilation	3	16.7%	0	0.0%	—	**0.042**
Prone position	2	11.1%	0	0.0%		0.125
Renal impairment	2	11.1%	0	0.0%		0.125
Maternal death	3	16.7%	0	0.0%		**0.042**
SARS‐CoV‐2 infection moment						
1st trimester	1	5.6%	5	15.6%	—	0.213
2nd trimester	3	16.7%	10	31.3%		
3rd trimester	14	77.8%	15	46.9%		
Puerperium	0	0.0%	2	6.3%		
Maternal outcomes						
Time between symptoms onset and laboratory diagnosis						
<7 days	14	77.8%	29	90.6%	0.36 (0.1–1.8)	0.566
≥7 days	4	22.2%	3	9.4%		
	(*n =* 18)^a^		(*n =* 29)^a^			
Delivery route						
C‐section	13	72.2%	17	58.6%	1.84 (0.5–6.5)	0.346
Vaginal	5	27.8%	12	41.4%		
Preterm birth (<37 weeks)	9	50.0%	8	27.6%	2.63 (0.8–9.0)	0.120
Preeclampsia	3	16.7%	4	13.8%	1.25 (0.25–6.37)	1.000
Pregnancy and perinatal outcomes	(*n =* 20)^b^		(*n =* 32)^b^			
Miscarriage/abortion/ectopic pregnancy	2	10.5%	1	3.1%	3.44 (0.29–40.71)	0.301
	(*n =* 18)^c^		(*n =* 31)^†^			
Stillbirth	0	0.0%	0	0.0%	—	1
Neonatal death	0	0.0%	2^e^	6.5%		0.271
Live birth	18	100%	29	93.5%		0.279
Congenital anomaly	0	0.0%	5^f^	16.1%		0.072
	(*n =* 18)^d^		(*n =* 29)^d^			
Small for gestational age	4	22.2%	10	34.5%	0.57 (0.2–2.2)	0.412
Apgar score 5 min <7	4	22.2%	3	10.3%	5.39 (0.9–31.5)	0.086

*Note:* Bolded *P*‐values indicate statistical significance (*P*‐value < 0.05).

^a^ Cases with childbirth at Unicamp.

^b^ Fetuses of the cases with childbirth at Unicamp.

^c^ All live fetuses of the cases until delivery at Unicamp.

^d^ All live neonates of the cases with delivery at Unicamp.

^e^ Neonatal death by Body Stalker series (1) and anencephaly/multiple malformations (1).

^f^ Fetuses with Body Stalker series (1), cardiopathy (1), fetal adnexal cyst (1), anencephaly/multiple malformations (1), and asymmetric severe ventriculomegaly (1).

The findings showed significant higher maternal clinical severity in VOC cases (Table 2). The proportion of individuals with O_2_ saturation < 95% (odds ratio 24.8 [CI 2.7–223.3], *P*‐value <0.001) hospitalization (odds ratio 6.7 [CI 1.7–25.2], *P*‐value = 0.003), intensive care unit (ICU) admission (odds ratio 15.5 [CI 1.7–142.6], *P*‐value = 0.0036), incidence of invasive ventilation (*P*‐value = 0.042), and maternal death (*P*‐value = 0.042) were higher in the VOC group than in the non‐VOC group. (Table 2). Two out of three maternal death cases presented comorbidities, including diabetes (2/3), chronic arterial hypertension (2/3), and obesity (1/3). All three maternal death cases had preterm births prior to the death event. One case presented early‐onset preeclampsia at 24 weeks of gestation, concomitant with acute SARS‐CoV‐2 infection, leading to labor induction and subsequent neonatal death. The other two cases had medically indicated preterm births, with live‐born infants by c‐section at 29 and 30 gestational weeks prior to maternal death. Logistic regression reinforced the severity findings described above, showing that O_2_ saturation < 95% (odds ratio 24.8 [CI 2.75–223.30], *P*‐value = 0.004), hospitalization (odds ratio 6.7 [CI 1.8–25.2], *P*‐value = 0.003), and ICU maternal admission (odds ratio 15.50 [CI 1.7–142.6], *P*‐value = 0.016) were factors independently associated with SARS‐CoV‐2 VOCs (Table S4). In addition, using 70% of the total cases and the clinical data of desaturation, hospitalization, ICU admission, and maternal death for training the decision tree model, the analysis showed that hospitalization was able to predict a 62% probability that the patient was infected by a VOC lineage. Meanwhile, if the patient did not need hospitalization, there was an 89% probability of non‐VOC infection (Figure S2). The tree model was validated with the remaining 30% of cases, and it showed 66% accuracy (95% CI 38% to 88%), 62% sensitivity, and 71% specificity to detect VOC and non‐VOC cases in the considered population.

**FIGURE 1:**
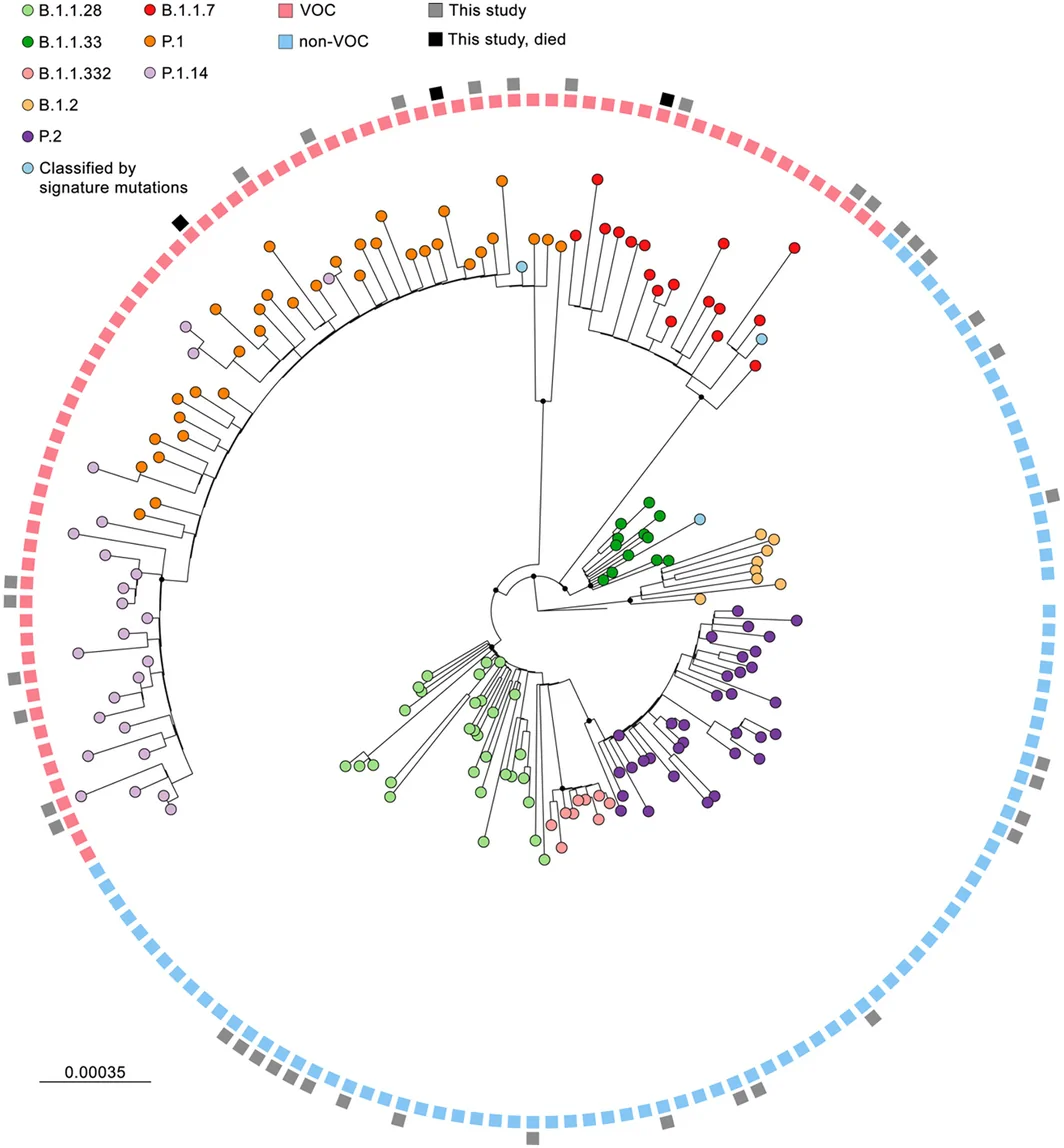
Maximum‐likelihood phylogenetic tree of sequences in this study (*n =* 18 variants of concern [VOCs] and *n =* 23 non‐VOCs) and representative sequences from GISAID (*n =* 130). Colors of terminal leaf nodes indicate lineages of SARS‐CoV‐2 according to Pangolin classification. Metadata is represented as colored squares indicating VOC or non‐VOC lineage and sequences obtained in this study. Black dots indicate the main nodes with ML bootstrap values >70% based on 1000 bootstrap replicates. The scale bar indicates the evolutionary distance in numbers of substitutions per nucleotide site. Sequences are available on GISAID according to numbers represented in Table S1.

From the 50 patients' samples, we identified six samples that were classified as Gamma (P.1), six samples as Gamma‐plus (P.1.14), and three samples as Alpha (B.1.1.7) (Tables S3 and S4). We also identified three samples with coverage less than 70%, which we could classify as Gamma (61.4% and 55% coverage) and Alpha (45.4% coverage). To construct an ML phylogenetic tree, we included 130 representative sequences, all 18 VOC sequences (including the three samples with less than 70% coverage), and 23 non‐VOC representative sequences from this study. ML analysis showed that all VOC sequences from this study were placed into the VOC branch, which included Gamma and Alpha VOCs (Figure 1). Non‐VOC sequences from our study were placed into the non‐VOC branch that included the B.1.1.28, B.1.1.33, B.1.2, B.1.1.332, and P.2 lineages (Figure 1).

**FIGURE 2:**
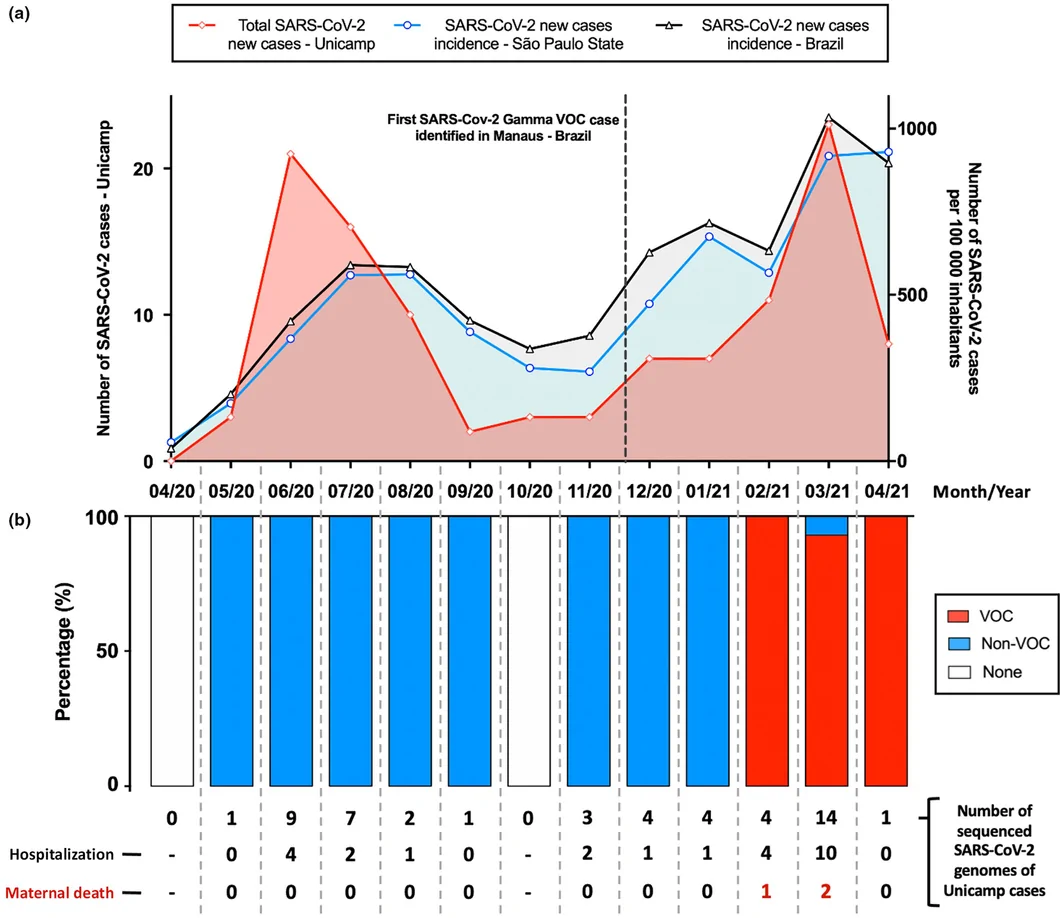
Temporal dispersion of the incidence of SARS‐CoV‐2 infection cases in Brazil and in São Paulo state parallel with the number of cases at Unicamp and their genomic characterization by month/year from May 2020 to April 2021. Each point on the scatter plot in the upper panel represents the number of cases at Unicamp (red) or the incidence per 100 000 inhabitants in the State of São Paulo (blue) and Brazil (black). In the percentage bar graph in the lower panel, each month has a bar displaying the classification of VOC referring to cases with the viral genome sequenced at Unicamp. Below each month of the bar chart are arranged from top to bottom: number of cases sequenced; hospitalizations number; and deaths number.

By analyzing the cases by month relative to COVID‐19 case incidence per 100 000 inhabitants in Brazil and São Paulo state, and the number of cases accumulated per month at Unicamp (Figure 2a), it was possible to observe an association between the magnitude of incidence/number of cases and the different virus variants at Unicamp (Figure 2). The epidemiological graph shows two peaks of SARS‐CoV‐2 detection over a 1‐year period. The first peak of cases in Unicamp (May 2020 to August 2020) was associated with the circulation of non‐VOCs. In contrast, the second peak (February 2021 to April 2021) was associated with the circulation of VOC: Alpha B.1.1.7 (B.1.1.7) and Gamma (both P.1 and P.1.14) (Figure 2b). Before VOC circulation, we did not report any maternal deaths. During VOCs circulation, there was an increase in the hospitalization rate of the sequenced cases as well as the emergence of the first cases of maternal death, one of which was registered in February and two in March. The three cases of death refer to the cases of the Gamma (two) and Alpha (one) VOCs.

## 4 DISCUSSION

This study provided an overview of the impact of different variants of SARS‐CoV‐2 infection, including VOCs, on SARS‐CoV‐2‐unvaccinated pregnant and postpartum individuals before COVID‐19 vaccination campaigns. Remarkably, our data indicate that clinical severity is increased in cases infected by VOCs (Alpha and Gamma), as indicated by the higher risk of oxygen desaturation, hospitalization, ICU admission, and rates of intubation and maternal death. We emphasize that before VOC circulation in the geographical area analyzed, there were zero maternal deaths in this referral maternity.

Our findings coincide with the increased morbidity and mortality of unvaccinated pregnant and postpartum individuals due to COVID‐19 cases in Brazil.^14^, ^17^ In 2020, maternal mortality was already of great concern; however, this scenario worsened radically in the beginning of 2021, concomitantly with the emergence of VOCs and their spread throughout Brazil. Relating our data with those from the official Brazilian national surveillance system^14^ on COVID‐19 severe acute respiratory syndrome (SARS) reported cases in pregnant/postpartum women and considering dates matching the beginning and end of this study and separating by the emergence of VOCs in Brazil with an estimated date (December 6, 2020),^16^ a strong difference in the national scenario was noticed. The ICU admission rate soared from 22.6% (1385 cases) to 48.69% (1840 cases), and mortality increased from 6.4% (392 maternal deaths) to 21.04% (795 maternal deaths).^14^ On a smaller scale, nevertheless with similar characteristics, these lethality data were reflected at the study referral center, strikingly with three maternal deaths in the first 4 months of 2021, all related to VOCs. Although the decision tree model had moderate accuracy, sensitivity, and specificity, it reinforces that hospitalization is more closely related to SARS‐CoV‐2 VOCs in pregnant women in this study.

As the obstetric population is a risk group for increased morbidity and mortality compared to the general population,^17^, ^18^ SARS‐CoV‐2 genomic variants have raised questions on their role in increasing (or not) the risk of adverse outcomes.^19^, ^20^ Studies indicate that the Delta VOC‐dominant period involves more severe COVID‐19 and worse maternal outcomes compared to previous cases, particularly in the low‐vaccine‐acceptance pregnant population.^21^, ^22^ An increased risk of hospitalization and ICU admission were also associated with reported cases of SARS‐CoV‐2 Alpha and Beta VOCs.^19^ Other SARS‐CoV‐2 VOCs emerged after the first half of 2021 and replaced the preponderant circulation of Gamma in Brazil and Delta globally.^4^, ^23^, ^24^ Since its introduction in the final quarter of 2021, the highly mutated Omicron variant and its subvariants have consistently dominated global cases.^25^, ^26^, ^27^ However, even with the replacement and dynamics of the circulating variant from a local and global perspective, to date, no study has directly linked Gamma VOC with higher adverse maternal outcomes and mortality, with analysis focusing only on the temporal aspect of the incidence of infected cases.^13^, ^21^, ^22^

Vaccination of pregnant and postpartum women against SARS‐CoV‐2 began in May 2021 in Brazil.^14^, ^28^ We highlight that the timeframe of population assessment in our study preceded the implementation of COVID‐19 vaccination for pregnant women, thus precluding the inclusion of vaccinated individuals. Studies have demonstrated that vaccination during pregnancy and the postpartum period induces a robust immune response to SARS‐CoV‐2 and is associated with reduced maternal clinical severity.^11^, ^12^, ^13^

Unicamp is a referral maternity hospital with known high‐quality clinical management and high‐risk care that enables improvement on clinical outcomes compared to non‐referral services available, especially during the COVID‐19 pandemic in Brazil. The characteristics of Unicamp clinical management indicate that the adverse outcomes described in this study associated with VOC infection were normalized in terms of quality of the health care offered to the study participants. Studies suggest that VOC impacts are associated with pre‐existing geographic inequities, transient infection peaks, and concomitant breakdown in high healthcare demand.^29^, ^30^

This study has several limitations. First, it was performed in a single‐center cohort, tracing a specific geolocated population, mostly within a medium‐to high‐density urban area. Second, the cases selected for sequencing had a Ct limit lower than 28, which was a limiting factor for sampling and restricting the sample size. The maternal and perinatal outcomes reported are most likely not generalizable, given the high prevalence of comorbidities and the availability of high‐complexity care infrastructure characteristic of this referral maternity center. While clinical variables were broadly captured, some limitations remain, including the lack of detailed information on specific indications for cesarean delivery, such as whether the procedure was performed upon maternal request. The present analysis focused on upper respiratory tract samples evaluation for SARS‐CoV‐2 genome variant classification, although diverse biological samples were collected from the same cohort, including placental samples for detailed morphological assessment and evaluation of focal placental damage due to SARS‐CoV‐2 infection.^31^, ^32^, ^33^ The number of cases might be limited for the analysis of maternal and perinatal outcomes; however, for genome sequencing and characterizing the impact of Gamma VOC, the sample size aligns with the translational approach prioritizing in‐depth analysis.

## 5 CONCLUSION

In summary, the present study indicates that maternal VOC SARS‐CoV‐2, mainly Gamma, increase the risk of desaturation, hospitalization, and ICU admission, intubation and maternal death. The increased risk of clinical severity in the unvaccinated obstetric population highlights the importance of addressing the repercussions of SARS‐CoV‐2 genomic evolution in all contexts, particularly in vulnerable populations without SARS‐CoV‐2 vaccination.

## AUTHOR CONTRIBUTIONS

G.M.N., F.G., J.L.P.M., and M.L.C. developed the study concept. G.M.N., J.P.S.G, A.G.L., G.J.L., C.C.R.V., G.A.P., K.C.S.K., A.Z.S., G.D.M., R.N.A., M.L.M., R.C.P., R.S., J.G.C., M.N.N.S. and M.L.C. implemented the clinical and sociodemographic data collection and biological sampling and adequate storage. G.M.N., F.G., M.R.A., J.F., and P.P.B. performed laboratory analyses and SARS‐CoV‐2 genome sequencing. G.M.N., F.G., L.S.M., D.A.T.T., P.P.B., W.M.S., and J.L.P.M performed genomic analyses. G.M.N., D.A.T.T., and J.P.S.G performed statistical analyses. G.M.N. wrote the first draft of the manuscript and all authors further reviewed the manuscript. J.L.P.M. and M.L.C. supervised project analysis. R.C.D., R. S., J.G.C., R.G.S., M.N.N.S., F.R.S., E.C.S., N.R.F., W.M.S., J.L.P.M., and M.L.C. coordinated the study and were responsible for the project administration and funding acquisition. Collaboration between all authors was fundamental for biological sampling, clinical data collection, and work development. All the authors read and agreed to the published version of the manuscript.

## FUNDING INFORMATION

This study was supported by *Fundo de Apoio ao Ensino, à Pesquisa e à Extensão* (FAEPEX‐Unicamp) (grant numbers 2300/20 and 2266/20), by *Conselho Nacional de Desenvolvimento Científico e Tecnológico* (CNPq) (grant number 408407/2021‐2), by São Paulo Research Foundation (FAPESP) (grant number 2016/00194‐8, 2020/04558‐0 and 2018/14372‐0); by the Brazilian Ministry of Science, Technology, and Innovation (MCTI), through the *Rede Corona‐ômica* funded by the Financier of Studies and Projects (FINEP) (grant number 01.20.0003.00), by Rede Vírus/MCTI (FINEP grant number 01.20.0029.000462/20) and by the Medical Research Council and FAPESP‐Brazil‐UK Center for (Arbo) virus Discovery, Diagnosis, Genomics, and Epidemiology partnership award (MR/S0195/1 and FAPESP 2018/14389‐0). G.M.N. was supported by *Coordenação de Aperfeiçoamento de Pessoal de Nível Superior* (CAPES) (grant numbers 88887.600190/2021‐00 and 88887.712761/2022‐00). D.A.T.‐T. and L.S.M. were supported by CNPq fellowships (grant numbers 141844/2019‐1 and 382206/2020‐7). M.R.A., P.P.B., and J.F. are recipients of scholarships provided by CAPES (grant numbers 88887.356527/2019‐00, 88887.661921/2022‐00 and 88887.513498/2020‐00). N.R.F. was supported by a Wellcome Trust and Royal Society Sir Henry Dale Fellowship (grant number 204311/Z/16/Z). WMdS was supported by a Global Virus Network Fellowship. J.L.P.‐M. was supported by CNPq (grant number 305628/2020‐8).

## CONFLICT OF INTEREST STATEMENT

The authors have no competing interests to declare.

## Supporting information


Figures S1–S2.
Tables S1–S5.

## Data Availability

All SARS‐CoV‐2 genome sequences generated in this study and all representative sequences are available from the GISAID Initiative database (www.gisaid.org/), accession IDs list in Table S1. Regarding the clinical and sociodemographic participants' data, the REBRACO Study Group is still conducting other analyses, and the ethical approval given for the study did not consider the public availability of the information. The data will be available upon request and under revision by the Ethical Review Board.
